# Serotonin differentially modulates the temporal dynamics of the limbic response to facial emotions in male adults with and without autism spectrum disorder (ASD): a randomised placebo-controlled single-dose crossover trial

**DOI:** 10.1038/s41386-020-0693-0

**Published:** 2020-05-10

**Authors:** Nichol M. L. Wong, James L. Findon, Robert H. Wichers, Vincent Giampietro, Vladimira Stoencheva, Clodagh M. Murphy, Sarah Blainey, Christine Ecker, Declan G. Murphy, Grainne M. McAlonan, Eileen Daly

**Affiliations:** 1 Department of Forensic and Neurodevelopmental Sciences, Institute of Psychiatry, Psychology and Neuroscience, King’s College London, London, UK; 2 Sackler Institute for Translational Neurodevelopment, Institute of Psychiatry, Psychology and Neuroscience, King’s College London, London, UK; 3 Biomedical Research Centre for Mental Health at the Institute of Psychiatry, Psychology and Neuroscience and South London and Maudsley NHS Foundation Trust, London, UK; 4 Department of Psychology, Institute of Psychiatry, Psychology and Neuroscience, King’s College London, London, United Kingdom; 5 Behavioural Genetics Clinic, Adult Autism and ADHD Service, Behavioural and Developmental Psychiatry Clinical Academic Group, South London and Maudsley Foundation NHS Trust, London, UK; 6 Department of Neuroimaging, Institute of Psychiatry, Psychology, and Neuroscience, King’s College London, London, UK; 7 Department of Child and Adolescent Psychiatry, Psychosomatics and Psychotherapy, Goethe University Frankfurt am Main, Frankfurt, Germany; 8 MRC Centre for Neurodevelopmental Disorders, King’s College London, London, UK

**Keywords:** Limbic system, Neurochemistry

## Abstract

Emotion processing—including signals from facial expressions—is often altered in individuals with autism spectrum disorder (ASD). The biological basis of this is poorly understood but may include neurochemically mediated differences in the responsivity of key ‘limbic’ regions (including amygdala, ventromedial prefrontal cortex (vmPFC) and nucleus accumbens (NAc)). Emerging evidence also suggests that ASD may be a disorder of brain temporal dynamics. Moreover, serotonin (5-HT) has been shown to be a key regulator of both facial-emotion processing and brain dynamics, and 5-HT abnormalities have been consistently implicated in ASD. To date, however, no one has examined how 5-HT influences the dynamics of facial-emotion processing in ASD. Therefore, we compared the influence of 5-HT on the responsivity of brain dynamics during facial-emotion processing in individuals with and without ASD. Participants completed a facial-emotion processing fMRI task at least 8 days apart using a randomised double-blind crossover design. At each visit they received either a single 20-mg oral dose of the selective serotonin reuptake inhibitor (SSRI) citalopram or placebo. We found that citalopram (which increases levels of 5-HT) caused sustained activation in key limbic regions during processing of negative facial emotions in adults with ASD—but not in neurotypical adults. The neurotypical adults’ limbic response reverted more rapidly to baseline following a 5-HT-challenge. Our results suggest that serotonergic homoeostatic control of the temporal dynamics in limbic regions is altered in adults with ASD, and provide a fresh perspective on the biology of ASD.

## Introduction

Autism spectrum disorder (ASD) is a neurodevelopmental disorder with a worldwide prevalence rate of ~1% [1]. It is characterised by restricted and repetitive patterns of behaviour, impaired social interaction and communication, and atypical sensitivity to sensory stimuli [2]. Specifically, the impairment in social interaction is thought to be at least partly underpinned by altered processing of facial emotions [3].

Key components of the limbic system implicated in human social behaviour and emotion processing include the amygdala, the nucleus accumbens (NAc), and the ventromedial prefrontal cortex (vmPFC) [4]. For instance, in the neurotypical population, the amygdala has been robustly implicated in the processing of facial emotions and is closely linked to processing of fear, anger [5] and other emotional perceptions [6, 7] experienced explicitly or passively [8]. In contrast, the NAc—through its dense interconnections with amygdala [9]—facilitates motor behaviours driven by emotionally salient stimuli [10]. Furthermore, there is consensus that vmPFC connections to the amygdala and NAc have a regulatory role in emotion and social cognition [11]. Alterations in the function of these major limbic regions have been reported in ASD, however, findings have been inconsistent. For example, prior studies have described hypo- and hyper-activity (or even no change in activation) during facial-emotion processing within amygdala [12–18], striatum [13, 15] and vmPFC [14, 19]. Some of the inconsistency in findings is likely to be caused by differences between the experimental methods used and the sample characteristics (e.g., age).

In addition, however, previous work has mostly examined group differences in average brain activation across a fixed time period—and this may not optimally capture brain activity. The brain is in constant flux [20, 21] and so it may be better to capture fluctuations in brain activity over time. This may be especially important here as there is emerging evidence that the temporal dynamics of brain activity are different in ASD. For instance, during resting-state fMRI, overly ‘stable’ brain dynamics have been reported in individuals with ASD as compared with neurotypical individuals [22]. Therefore, the analysis of average activations across the entire time period of the fMRI contrast of interest may mask crucial group differences in temporal dynamics. Support for this hypothesis comes from preliminary studies of amygdala ‘habituation’ during fMRI tasks (defined as the progressive decrease in response to repeatedly presented stimuli). Habituation of amygdala activation in response to facial emotions has been reported to be altered in ASD [23] relative to neurotypical controls [24] during both explicit [25] and implicit [26, 27] fMRI paradigms. Yet, the biological basis of the habituation phenomenon in ASD is still largely unknown.

The regulation of brain temporal dynamics including habituation is likely to involve co-ordinated action of multiple neuro-signalling systems [28, 29]. Among these, the serotonin (5-HT) system is likely to be especially important. For instance, infusion of psilocybin, which has potent psycho-active effects at the 5-HT_2A_ receptor, has been reported to impact on frontoparietal network dynamics in neurotypical individuals [30]. Stimulation of 5-HT_2A_ receptors could increase the excitability of the pyramidal neurons where the receptors are highly expressed and subsequently modulate the widespread neural systems [31]. Moreover, differences in 5-HT pathways are some of the most consistent findings in ASD [32]. Elevated whole-blood serotonin has been recorded in around a third of children and adults with ASD [33, 34], and ASD has been strongly linked to 5-HT genetic polymorphisms and candidate genes within 5-HT pathways [35–37]. Also, there is preliminary evidence for differences in 5-HT_2A_ receptor density in the ‘social brain’ (including anterior cingulate cortex) of adults with ASD [38]. Furthermore, using a tryptophan depletion protocol, we have previously found that lowering 5-HT levels has opposing effects on brain activation during processing of facial emotions in individuals with and without ASD [17]. In summary, 5-HT is a key regulator of facial-emotion processing and brain temporal dynamics [30, 39–42], which are both altered in ASD [3, 32, 33, 43]. However, nobody has directly examined how 5-HT influences the dynamics of facial-emotion processing in ASD.

Therefore, in this study we tested the hypothesis that the effects of 5-HT on the responsivity of brain dynamics of facial-emotion processing are different in individuals with and without ASD. Different aspects of brain dynamics, including the time-varying connectivity and time-frequency coherence [44], have been studied in ASD [22, 45, 46]. Here we were interested in the brain dynamics as measured by fMRI activations during processing of repeating facial-emotional stimuli [25–27, 47]. We conducted a single oral-dose pharmacological-fMRI study, and examined how the habituation pattern during baseline (i.e., placebo) would be changed by citalopram, a selective serotonin reuptake inhibitor (SSRI), administered in a double-blind, randomised order during a facial-emotion processing task. We focussed on the major limbic regions of interest (ROIs), namely the amygdala, vmPFC and NAc.

## Materials and methods

### Participants

We included 40 right-handed adult males (19 with ASD and 21 neurotypical controls, 18–60 years old), which the sample size is comparable to the previous fMRI study [17]. Participants with ASD were recruited through the National Adult Autism Service at the Maudsley Hospital and had a clinical diagnosis of ASD made by the multidisciplinary specialist team following ICD-10 research diagnostic criteria [48] and the Autism Diagnostic Observation Schedule (ADOS) [49] were completed for ASD participants where possible. For individuals with and without ASD, we only included individuals with no history of major medical disorders that could influence cognitive performance, other major mental illnesses, genetic disorders associated with ASD, alcohol or substance dependence, and who were not taking any medication affecting the 5-HT system. All participants underwent the Wechsler Abbreviated Scale of Intelligence test [50] to ensure that we only included those with an IQ of >70. Participants’ behaviours including social traits related to autism, namely ‘autistic traits’, were measured by autism-spectrum quotient [51]. Participants’ anxiety and depression were measured by Hamilton Anxiety Rating Scale (HAM-A) [52] and Hamilton Depression Rating Scale (HAM-D) [53], respectively. All participants gave written, informed consent after receiving a complete description of the study. The study was ethically approved by the Stanmore NHS Research Ethics Committee (reference: 14/LO/0663).

### Drug administration and study procedures

This study was conducted at the Institute of Psychiatry, Psychology and Neuroscience at De Crespigny Park, SE5 8AF, London, UK (December 2014 to December 2016). It adopted a placebo-controlled, randomised, double-blind, repeated-measures, crossover case-control study design as part of a larger investigation into the brain response to 5-HT medications in ASD (clinicaltrials.gov identifier: NCT04145076). Placebo and citalopram were allocated in a pseudo-randomised order, approximately half in each group attended a placebo visit before citalopram and the other half attended a citalopram visit before placebo. This random allocation was administered by GMM (https://www.random.org/) and both participants and researchers were blinded to the allocation. Participants were asked to complete two scanning visits, separated by at least 8 days to allow for complete washout of the drug. Participants were given either an acute single dose of encapsulated citalopram (20 mg), or encapsulated placebo (ascorbic acid) 3 h before each scanning session, as citalopram reaches its peak plasma level after ~3 h [54]. All the scanning sessions were completed within 1.5 h to ensure that citalopram did not reach half-life during scanning [55]. All participants received pre- and post-scan medical screening by a physician after administration of both drugs (placebo and citalopram).

### Facial-emotion processing fMRI task

**Fig. 1:**
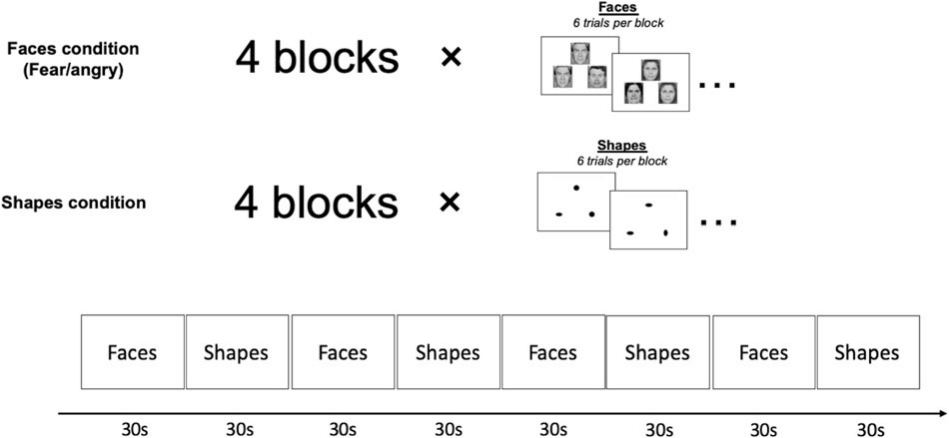
The design of the facial-emotion processing task. The task had four blocks of trials with fearful or angry faces and four blocks of trials with geometric shapes. There were six trials in each block and each trial was 5-s long. Participants were asked to indicate which image in the lower panel was identical to the target image in the upper panel in each trial. Each trial was presented twice throughout the task and the faces were sex and emotion balanced.

An adapted version of a face-matching task [5] was administered to participants during each scanning session. The task consisted of four blocks of faces and four blocks of geometric shapes, with six 5-s trials per block (Fig. 1). In each trial, participants were asked to indicate, by pressing buttons on a response box with their right hand, which of the two images in the lower panel were identical to the target image in the upper panel. Each trial was presented twice. Half of the faces were ‘angry’ and the other half were ‘fearful’. All faces were balanced for sex.

### MRI data acquisition and preprocessing

The fMRI data were acquired on a 3T General Electric Signa HD × Twinspeed scanner (Milwaukee, Wisc.) fitted with a quadrature birdcage head coil: TR = 2000 ms, TE = 30 ms, FOV = 192 × 192 mm, voxel size = 3 × 3 × 5 mm, flip angle = 80°, number of time points = 135. T1-weighted structural data was acquired sagittally: TR = 7.312 ms, TE = 3.016 ms, FOV = 270 × 270 mm, matrix size = 256 × 256, slice thickness = 1.2 mm, flip angle = 11°.

Each participant’s fMRI images were first corrected for slice-timing and head movement with FSL [56, 57]. Volumes in each subject that had framewise displacement (FD) >0.5 mm [57] were identified using *fsl_motion_outliers* and were subsequently regressed out in the first-level general linear model (GLM) in FSL [56, 57]. A temporal high-pass filter at 128 s was applied and the fMRI data were smoothed by 8 mm full width at half-maximum kernel. One ASD participant had more than 30% volumes with FD > 0.5 mm and was removed from further analyses, giving us a final sample of 39 individuals.

### ROI selection

**Fig. 2:**
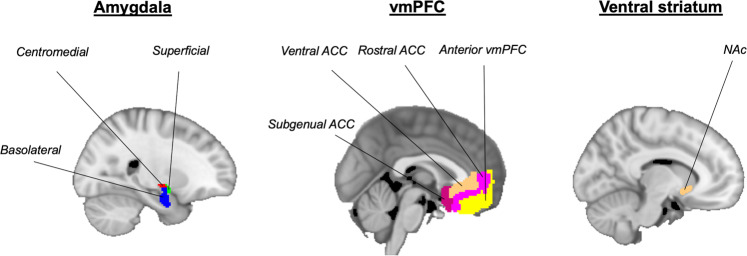
Regions of interest. The amygdala regions of interest (ROIs) included the basolateral amygdala (BLA), centromedial amygdala (CMA) and the superficial amygdala (SFA). The ventromedial prefrontal cortex (vmPFC) ROIs included the subgenual anterior cingulate cortex (sgACC), the rostral ACC (rACC), the ventral ACC (vACC) and the anterior vmPFC (avmPFC). The nucleus accumbens (NAc) were also included as ROIs.

We specifically focused on the amygdala, vmPFC and NAc based on hypotheses derived from previous work [4]. The amygdala ROIs were derived from FSL’s Juelich histological atlas [58] thresholding at 50%, and each voxel was assigned to one ROI, consistent to previous studies [59]. Our amygdala ROIs included the centromedial amygdala, basolateral amygdala (BLA) and the superficial amygdala (SFA). The vmPFC ROIs were derived from the vmPFC atlas of asymmetric and probabilistic cytoarchitectonic maps [60], and included the subgenual anterior cingulate cortex (sgACC), the rostral ACC (rACC), the ventral ACC (vACC) and the anterior vmPFC (avmPFC) [59] (Fig. 2). The NAc ROIs were defined according to FSL’s Harvard-Oxford subcortical structural atlas [61].

### Habituation of functional activation to negative facial emotions

**Fig. 3:**
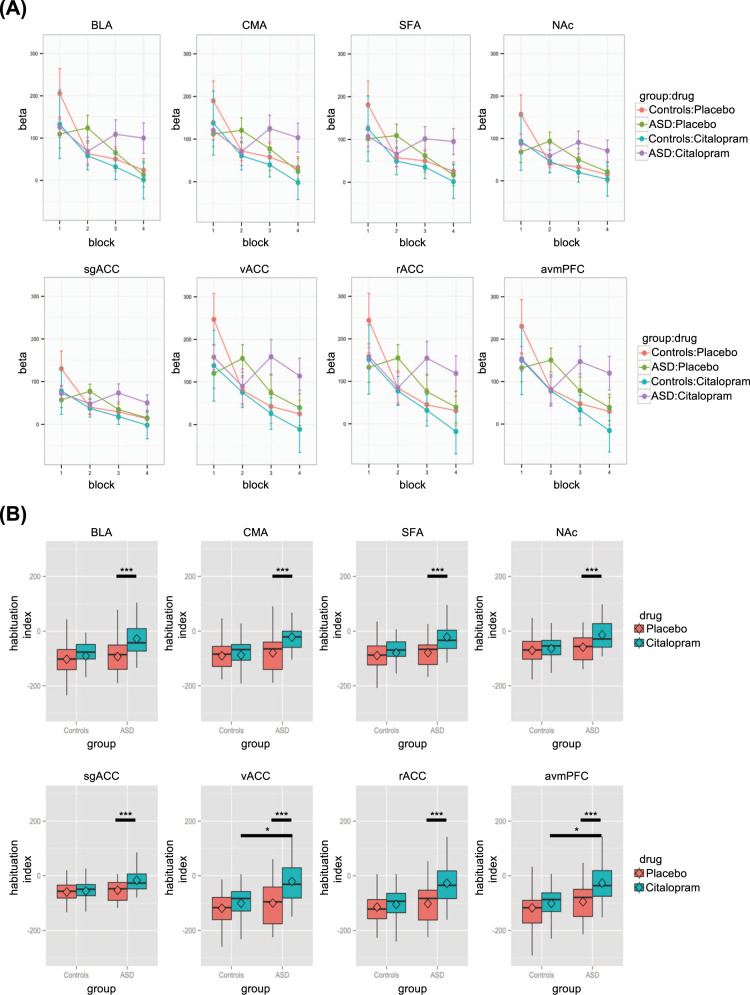
Habituation to negative facial emotions. **a** The block-wise beta of *Faces>Shapes* within the ROIs of controls and individuals with autism spectrum disorder (ASD) for the two drug conditions (placebo and citalopram) were extracted and here the means are plotted with standard error bars. **b** Using a regression approach to summarise the decrease in activations across time (i.e., habituation), absolute habituation indices were obtained and significant group × drug interaction effects on absolute habituation indices could be observed in all ROIs, including basolateral amygdala (BLA), centromedial amygdala (CMA), superficial amygdala (SFA), nucleus accumbens (NAc), subgenual anterior cingulate cortex (sgACC), ventral ACC (vACC), rostral ACC (rACC) and anterior ventromedial prefrontal cortex (avmPFC) (*p* corrected < 0.001). Significance for post-hoc analyses corrected for multiple comparisons is demonstrated with asterisks. *p < 0.05; ***p < 0.001.

To investigate the habituation of neural response to facial emotions, the first-level GLM analysis for each subject was conducted at voxel-level across the whole brain with one regressor per block of faces or shapes in addition to the six motion regressors derived from the correction procedure of motion artefact (i.e., 14 regressors in total). The block regressors modelled the onset and duration of each block and were convolved with the canonical hemodynamic response function. Each block was contrasted to the mean of all remaining blocks to scale the output block-wise beta to the overall mean [23]. The output beta maps were then normalised to standard space through registering to their skull-stripped structural data. All the fMRI images were re-sampled to a voxel size of 2 × 2 × 2 mm. The average block-wise beta of *Faces>Shapes* within each ROI in each subject for their two drug conditions were then extracted (Fig. 3a). As suggested by previous studies, an absolute habituation index is a reliable way to characterise the decrease in fMRI activation across blocks correcting for the initial activation [23]. This was obtained by regressing the block-wise beta on the logarithm of block numbers. Based on the regression,

$$Y = bX + a$$

the average beta of an ROI of each block in each participant (*Y*) was set up to be predicted by the logarithm of block numbers (*X*) (i.e., ln(1) = 0, ln(2) = 0.69, ln(3) = 1.10, ln(4) = 1.39). The logarithm of block numbers was adopted because habituation might not have a linear profile and logarithm transform has been reported to allow a better model fit [23, 62]. This also allows comparison with previous studies that used the same approach [23, 27]. As (*b)* (the change of activation of the ROI across blocks) was dependent on (*a)* (the estimated initial activation), an absolute habituation index of the ROI (*b′*) was further obtained,

$$b^\prime = b-c\left( {a - \overline a } \right)$$

by accounting for the slope (*c*) of (*b)* on (*a)* and the mean (*ā*) of (*a)*.

### Statistical analyses

All the analyses of demographics, autism traits, anxiety & depression levels and behavioural task performances were performed with *t*-tests and mixed-design analysis of variance (ANOVA) using *t.test* and *ezANOVA* respectively in *R* (https://www.r-project.org). Significance was inferred when *p* < 0.05.

For the task-fMRI data, exploratory whole-brain voxel-wise analysis on the functional activations investigating group × drug interaction effects in the contrast *Block 1*_*Faces* *>* *Shapes*_ *>* *Block 4*_*Faces* *>* *Shapes*_ (i.e., difference in *Faces* *>* *Shapes* functional activations between block 1 and block 4) were performed to explore whether any differences in the habituation profile observed were a generalised ‘whole-brain’ phenomenon. Please refer to Supplementary Information for details and results.

We focused our investigation on the functional activation within our hypothesised ROIs. Group × drug interaction effects on the absolute habituation indices for the contrast *Faces* *>* *Shapes* were compared between individuals with and without ASD in the placebo and citalopram conditions, tested by linear mixed-effect models using *lmer* in *R* (https://www.r-project.org). We investigated whether the absolute habituation indices were modulated by group and drug conditions while accounting for the random intercept for each participant in the linear mixed-effect models fitted by maximum likelihood for model comparisons. As laterality was not the focus of the current study, hemisphere (left vs. right), in addition to age, IQ, anxiety and depression, were included as covariates in all the linear mixed-effect models. *P* values for effects were estimated by likelihood ratio tests between models and were corrected (*p*_corrected_) for the eight subregions. Post-hoc pairwise tests corrected for multiple comparisons were performed. Significance was inferred when corrected *p* values < 0.05 and potential confounding effects of any differences in covariate measures were examined using regression.

We also collapsed the block-wise beta estimates across blocks into average beta estimates within each ROI and findings on the average beta estimates are reported in Supplementary Fig. 1.

## Results

### Demographics and clinical characteristics

There were no significant differences in age (*t*_36_ = 0.737, *p* = 0.466) and IQ (*t*_36_ = 0.774, *p* = 0.444) between individuals with ASD and neurotypical controls. As expected, individuals with ASD scored significantly higher in autism traits (*t*_28.2_ = 5.845, *p* < 0.001), anxiety (*t*_28.5_ = 2.863, *p* = 0.008) and depression levels (*t*_28.4_ = 3.281, *p* = 0.003) (Table 1). Only one individual with ASD and one control had anxiety scores ≥17, the clinical threshold of HAM-A and HAM-D. Both control and ASD groups had significantly lower mean scores than the clinical threshold of HAM-A and HAM-D, as revealed by one-sample *t*-tests (*t* ≥ 5.803, *p* < 0.001).

**Table 1:** Demographics and clinical characteristics of the study sample.

	ASD	Controls	*T*	*p*
	(*n* = 18)	(*n* = 21)		
Demographics				
Age (years)	29 (10)	27 (9)	0.737	0.466
Intelligence quotient	111 (17)	115 (10)	0.744	0.444
Clinical characteristics				
AQ	31 (11)	13 (7)	5.845	<0.001
HAM-A	8 (7)	3 (4)	2.863	0.008
HAM-D	7 (5)	2 (3)	3.281	0.003
ADOS-C	3 (2)	–		
ADOS-S	6 (2)	–		

The means are presented with standard deviations in parenthesis.

*HAM-A* Hamilton Anxiety Rating Scale; *HAM-D* Hamilton Depression Rating Scale, *ADOS-C*  Autism Diagnostic Observation Schedule–Communication domain, *ADOS-S*  Autism Diagnostic Observation Schedule–Reciprocal Social Interaction domain, *AQ*  autism-spectrum quotient.

### Task behavioural performance

A group × drug × stimuli factorial design was used to investigate the accuracy of the individuals’ responses during the task for the two drug conditions (placebo and citalopram), and no interaction effects were identified (*p* > 0.05). We only found a significant main effect of stimuli type—with individuals having higher accuracy in matching faces than shapes regardless of group (*F*_1,35_ = 45.893, *p* < 0.001) (Table 2). Similarly, no interaction effects were identified in the individuals’ response reaction time during the task (*p* > 0.05). A significant main effect of stimuli type was observed—with individuals matching faces slower than shapes regardless of group (*F*_1,35_ = 31.061, *p* < 0.001). Moreover, a significant main effect of group was observed where individuals with ASD responded more slowly than controls across all trials (*F*_1,35_ = 4.698, *p* = 0.037) (Table 2).

**Table 2:** Behavioural performance of controls and autistic adults during the task.

	Stimuli	Drug	Group	Mean (SD)
Accuracy (%)				
	Shapes	Placebo	Controls	93.54 (6.550)
			ASD	94.85 (5.419)
		Citaloparm	Controls	94.79 (5.036)
			ASD	94.85 (4.780)
	Faces	Placebo	Controls	97.92 (5.815)
			ASD	98.53 (4.151)
		Citaloparm	Controls	98.96 (2.985)
			ASD	99.51 (1.384)
*F*_Stimuli_	45.893***			
*F*_drug_		1.377		
*F*_group_			0.298	
*F*_stimuli × drug_	0.123			
*F*_stimuli × group_	0.007			
*F*_drug × group_	0.221			
*F*_stimuli __× drug × group_	0.291			
Reaction time (s)				
	Shapes	Placebo	Controls	0.9835 (0.19788)
			ASD	1.1374 (0.27225)
		Citaloparm	Controls	0.9945 (0.23690)
			ASD	1.0865 (0.27116)
	Faces	Placebo	Controls	1.0358 (0.20316)
			ASD	1.2636 (0.25622)
		Citaloparm	Controls	1.0803 (0.22423)
			ASD	1.2636 (0.25622)
*F*_stimuli_	31.061***			
*F*_drug_		0.048		
*F*_group_			4.698*	
*F*_stimuli × drug_	1.831			
*F*_stimuli × group_	3.539			
*F*_drug × group_	2.115			
*F*_stimuli × drug × group_	0.027			

The means, standard deviations (SD) and the statistics are presented.

*p < 0.05; ***p < 0.001.

### Group differences in habituation in ROIs

Using one-sample *t*-test, we found that there was habituation to negative facial emotions in all the ROIs for both controls and individuals with ASD in the placebo condition (*p*_corrected_ < 0.05). However, only controls exhibited habituation in the citalopram condition (*p*_corrected_ < 0.05). Habituation to negative facial emotions was not evident in any ROI for individuals with ASD in the citalopram condition (*p*_corrected_ ≥ 0.657). Using the mean absolute habituation indices of controls in each condition (baseline and citalopram) as references, the number of autistic adults having higher or lower habituation at baseline (placebo) and post citalopram are also reported (Table 3; Supplementary Fig. 2).

**Table 3:** Habituation characteristics of autistic adults comparing relative to control mean at baseline placebo condition and post citalopram.

	Placebo		Citalopram	
	Higher habituation	Lower habituation	Higher habituation	Lower habituation
	*n* (%)	*n* (%)	*n* (%)	*n* (%)
BLA	11 (61%)	7 (39%)	15 (83%)	3 (17%)
CMA	10 (56%)	8 (44%)	16 (89%)	2 (11%)
SFA	11 (61%)	7 (39%)	16 (89%)	2 (11%)
NAc	11 (61%)	7 (39%)	14 (78%)	4 (22%)
sgACC	11 (61%)	7 (39%)	15 (83%)	3 (17%)
vACC	11 (61%)	7 (39%)	17 (94%)	1 (6%)
rACC	11 (61%)	7 (39%)	17 (94%)	1 (6%)
avmPFC	11 (61%)	7 (39%)	17 (94%)	1 (6%)

*BLA* basolateral amygdala, *CMA* centromedial amygdala, *SFA* superficial amygdala, *NAc* nucleus accumbens, *sgACC* subgenual anterior cingulate cortex, *vACC* ventral ACC, *rACC* rostral ACC, *avmPFC* anterior vmPFC.

We also explored whether there were associations between habituation in the ROIs and the task behavioural performance in terms of the difference in accuracy and reaction time between matching faces and shapes, and found no significant associations (*p* ≥ 0.066).

To elucidate whether there was a significant difference between the responsivity to citalopram in individuals with and without ASD, we investigated whether group × drug interaction effects could be observed in our ROIs during habituation. All ROIs revealed significant interaction effects (*b* ≥ 31.285, *χ*^2^ ≥ 27.843, *p*_corrected_ < 0.001) (Fig. 3b). Post-hoc analyses revealed no group differences in placebo condition in any ROI (*p* > 0.05). On the contrary, significantly lower overall habituation (i.e., less negative indices) in ASD compared with controls was evident in the citalopram condition in avmPFC (*b* = 77.857, *χ*^2^ = 7.291, *p*_corrected_ = 0.042) and vACC (*b* = 84.339, *χ*^2^ = 7.064, *p*_corrected_ = 0.047). No differences between habituation in placebo and citalopram conditions were observed in controls (*p* > 0.05). Compared with the placebo condition, lower habituation (i.e., less negative indices) to negative facial emotions after citalopram intake was only detected in individuals with ASD in all ROIs (*b* ≥ 36.279, *χ*^2^ ≥ 90.001, *p*_corrected_ < 0.001).

### Associations between covariates and 5-HT induced habituation change

Autism traits, anxiety and depression scores were not associated with the change in habituation in any ROI across groups (*b* ≤ 4.028, *χ*^2^ ≤ 1.285, *p* ≥ 0.257). Also, no significant interaction effects between autism traits, anxiety and depression scores and groups were observed in any ROI (*b* ≥ −10.783, *χ*^2^ ≤ 2.314, *p* ≥ 0.128). Furthermore, the ADOS subscores (communication and social interaction) were not associated with the change in habituation in any ROI in individuals with ASD (*b* ≤ 2.145, *χ*^2^ ≤ 2.286, *p* ≥ 0.131).

## Discussion

We demonstrated (using a randomised double-blind crossover pharmacological-fMRI design) that the habituation of fMRI activation in key limbic regions during the processing of negative facial emotions was altered by 5-HT reuptake inhibition—but only in adults with ASD. In essence, in neurotypical adults, limbic response to emotional faces response reverted to baseline within 3 h following a 5-HT challenge but not in ASD. We suggest that this indicates altered homoeostatic control of limbic systems by 5-HT in ASD.

Previous fMRI investigations of amygdala habituation in response to sad [25], fearful [26] and neutral faces [25, 47] have generally reported that habituation (without pharmacological challenge) is reduced in ASD. Here we only observed a trend towards lower habituation in the key limbic regions to the emotional faces in ASD compared with controls at baseline (i.e., placebo condition), possibly due to differences in sample characteristics and the task adopted. For example, some previous studies investigated children and adolescents [25], whereas we recruited adults [63]. Previous pharmacological studies have found children are less likely to tolerate and/or respond to SSRIs with increased risk of adverse effects [64, 65], suggesting that there might be significant differences in brain 5-HT function in pre- and post-pubertal individuals with ASD [66]. We also used an adapted face-matching paradigm presenting a mix of angry and fearful faces [5] whereas previous studies used paradigms with fearful faces only [26], angry faces only [27] or included sad faces [25]. Thus, the studies differed in some key methodological aspects. Furthermore, we examined habituation across multiple blocks in the adapted face-matching paradigm, whereas previous studies were comparing the change in activations between two runs of their paradigms over a longer duration [25, 26]. Therefore, the habituation patterns captured in our study are broadly equivalent to the initial run of previous studies. Thus, the longer time-scale of previous studies precludes direct comparison of our approaches. It should also be emphasised that, due to the nature of the null hypothesis testing, the interpretation of our insignificant baseline differences should be considered with caution.

In our study, we found that citalopram (which increases levels of 5-HT) caused sustained activation in key limbic regions during processing of negative facial emotions in adults with ASD—but not in neurotypical adults. Although, the adults with ASD have higher anxiety and depression levels than the neurotypical adults in our study sample, the scores were significantly lower than the clinical threshold that a neurotypical individual with anxiety or depressive disorder would have. Nevertheless, given the group difference we also controlled for anxiety and depression scores in our statistical analyses to minimise any impact on our results and their interpretation. We did not observe effects of citalopram on task accuracy or response reaction time. We also did not observe any associations between task accuracy or response reaction time and habituation. Furthermore, we did not observe any associations between anxiety and depression scores and the effects of citalopram on habituation. Therefore, the effects of citalopram in ASD that we detected were not a consequence of more general drug effects on performance and/or presence of common co-occurring conditions. 5-HT has been strongly implicated in emotional behaviours [67] and manipulation of brain 5-HT levels has been shown to alter the processing and recognition of facial emotions [39–42]. Consequently, when abnormalities in the 5-HT system in individuals with ASD were identified [32, 33, 43] this was considered to be consistent with social behavioural difficulties. For example, reducing brain 5-HT using a tryptophan depletion paradigm ‘restored’ an fMRI pattern of case-control differences in ASD during facial-emotion processing to the baseline activation levels observed in neurotypical controls. This was a BOLD level response and we cannot be certain of the cellular mechanisms underlying it. It does however suggest that the brain activations generating the BOLD response in adults with and without ASD responded differently to the availability of 5-HT [17]. The present work takes this evidence for differential effects of 5-HT in ASD further and suggests that 5-HT has a differential impact on the brain temporal dynamics in people with and without ASD. This may reflect group differences in functioning of the 5-HT system and its relation to responsivity to modulation of 5-HT in individuals with ASD.

The cellular basis of this difference in responsivity to 5-HT is not known but may at least partly be genetic [35–37]. For example, reduced habituation of the amygdala response to facia emotion has been observed in individuals with ASD with low expression of the 5-HT transporter-linked promoter region, indicating that the altered facial-emotion processing in ASD could be genetically influenced in relation to the serotonergic system [68]. This fits with the suggestion that the 5-HT reuptake transporter protein, the major regulator of synaptic levels of 5-HT, is ‘overwhelmed’ or even impaired in individuals with ASD [69]. The end result is long-term impairment of serotonin transporter function which leads to permanently up- or down-regulated ancillary clearance uptake mechanisms for 5-HT and hence, altered homoeostasis of 5-HT in ASD [70]. We postulate that such a mechanism may contribute to the altered homeostasis of brain activity at the macro-scale level observed here.

A corollary of these findings is that we cannot assume that individuals with and without ASD respond to 5-HT drugs in the same way. While there is some evidence supporting the use of SSRIs for various indications in adults with ASD, the benefits are modest and especially in young people there appears to be an increased risk of adverse effects [64, 71]. Consensus guidelines for SSRI use in individuals with ASD currently state that they should be used in low doses and titrated up gradually with careful monitoring of side effects [71]. Whether the differential effect of SSRI on brain function observed here might contribute to a greater sensitivity and/or different clinical response to this family of medicines in ASD requires further investigation.

### Limitations

We acknowledge several limitations of this study. First, we did not have a large study sample size because we adhered to strict recruitment criteria with repeated testing and drug administration. However, we adopted a conservative statistical thresholding in controlling for multiple comparisons and because each participant provided both placebo and drug data, inter-individual variability was reduced. Future studies may include a larger sample. Second, the inclusion of only male participants limits generalisability of findings. However, it also limits potential sex differences in clinical profiles, pharmacology and functional activation in a modest sample size. Third, we used citalopram to investigate the modulatory effect of brain 5-HT levels and how brain activations might respond to a 5-HT challenge. Future studies could explore the receptor mechanisms underlying this finding with more selective drug probes of the 5-HT system. Fourth, we measured baseline anxiety and depression levels but could not measure again at the time of peak blood levels of citalopram when participants were in the scanner. Previous studies have shown that using a tryptophan depletion protocol to lower 5-HT levels in individuals with ASD would increase their anxiety levels [72]. Thus, the role of the 5-HT system in ASD is likely to be complex with effects depending on acute or chronic changes to 5-HT and the age of participants in any given study.

## Conclusions

We found that increasing levels of 5-HT using an SSRI caused a sustained activation in key limbic regions during processing of negative facial emotions in adults with ASD. In contrast the neurotypical response to emotion reverted to baseline. Thus, the homoeostatic control of serotonin pathways which regulate limbic system habituation is distinct in adults with ASD. Our results encourage a shift away from examining average differences in brain activity towards a focus on brain temporal dynamics. Our findings also indicate that we cannot assume that 5-HT drugs act in the same way in people with and without ASD. This study provides a fresh perspective on the biology of ASD and may help inform approaches to pharmacological intervention. It also encourages further examination of the clinical utility of altering habituation pharmacologically in ASD.

## Funding and disclosure

The authors acknowledge support from the Clinical Biochemistry Department at King’s College Hospital, the Maudsley Pharmacy Department and the Neurodevelopmental Clinic at the Maudsley Hospital.

The authors acknowledge support from the National Institute for Health Research (NIHR) Biomedical Research Centre for Mental Health at South London and Maudsley NHS Foundation Trust and Institute of Psychiatry, Psychology and Neuroscience King’s College London. This article presents independent research funded by the National Institute for Health Research (NIHR). The results leading to this publication has received funding from the Innovative Medicines Initiative 2 Joint Undertaking under grant agreement No 777394 for the project AIMS-2-TRIALS. This Joint Undertaking receives support from the European Union's Horizon 2020 research and innovation programme and EFPIA and AUTISM SPEAKS, Autistica, SFARI. The views expressed are those of the author(s) and not necessarily those of the IMI, NHS, the NIHR or the Department of Health. The authors declare no competing interests.

## Supplementary information

Supplementary Information

CONSORT Flowchart
