# Supplementary material for: Serotonin differentially modulates the temporal dynamics of the limbic response to facial emotions in male adults with and without autism spectrum disorder (ASD): a randomised placebo-controlled single-dose crossover trial
Source: Neuropsychopharmacology. 2020 May 10;45(13):2248–56. doi: 10.1038/s41386-020-0693-0 (PMC7784897; doi:10.1038/s41386-020-0693-0)
Supplement: Supplementary file 1 — Supplementary Information [file 41386_2020_693_MOESM1_ESM.pdf]

Serotonin differentially modulates the temporal dynamics of the limbic response to facial emotions in male adults with and without autism spectrum disorder (ASD): a randomised placebo-controlled single-dose cross-over trial

Nichol M.L. Wong, PhD[1,2,3,*], James L. Findon, PhD[1,2,4,#], Robert H. Wichers, PhD[1,2,5,#], Vincent Giampietro, PhD[6], Vladimira Stoencheva, BSc[1,5], Clodagh M. Murphy, MRCPsych[1,5], Sarah Blainey, DClinPsy[1,5], Christine Ecker, PhD[7], Declan G. Murphy, FRCPsych[1,2,3,8,#], Grainne M. McAlonan, PhD[1,2,3,5,8,#] and Eileen Daly, PhD[1,2,#]

[1]Department of Forensic and Neurodevelopmental Sciences, Institute of Psychiatry, Psychology and Neuroscience, King's College London, United Kingdom
[2]Sackler Institute for Translational Neurodevelopment, Institute of Psychiatry, Psychology and Neuroscience, King's College London, United Kingdom
[3]Biomedical Research Centre for Mental Health at the Institute of Psychiatry, Psychology and Neuroscience and South London and Maudsley NHS Foundation Trust, United Kingdom
[4]Department of Psychology, Institute of Psychiatry, Psychology and Neuroscience, King's College London, United Kingdom
[5]Behavioural Genetics Clinic, Adult Autism and ADHD Service, Behavioural and Developmental Psychiatry Clinical Academic Group, South London and Maudsley Foundation NHS Trust, UK
[6]Department of Neuroimaging, Institute of Psychiatry, Psychology, and Neuroscience, King's College London, United Kingdom
[7]Department of Child and Adolescent Psychiatry, Psychosomatics and Psychotherapy, Goethe University Frankfurt am Main, Germany
[8]MRC Centre for Neurodevelopmental Disorders, King's College London, United Kingdom

# Equal contribution

***Correspondence to:**
Nichol M.L. Wong, PhD
Department of Forensic and Neurodevelopmental Sciences
Institute of Psychiatry, Psychology and Neuroscience
King's College London
United Kingdom
Email: nichol.wong@kcl.ac.uk

Supplementary Information

Whole-brain analysis

For the task-fMRI data, exploratory whole-brain voxel-wise analysis on the functional activations investigating significant group×drug interaction effects in the contrast $Block1_{Faces>Shapes} > Block4_{Faces>Shapes}$ (i.e., difference in *Faces>Shapes* functional activations between block 1 and block 4) using *randomise* in FSL with 5000 permutations, controlling for age, IQ, anxiety, and depression were performed. The threshold-free-cluster-enhancement (TFCE) procedure was applied, adopting a family-wise-error (FWE)-corrected $p < 0.05$ to infer significance.

To elucidate whether there was a significant difference between the responsivity to citalopram in individuals with and without ASD, we explored whether group×drug interaction effects could be observed across the whole-brain during habituation. No significant group×drug interaction effects could be observed in the difference in *Faces>Shapes* functional activations between block 1 and block 4 ($p_{FWE} > 0.05$). There were also no significant group differences across the whole-brain in placebo and citalopram conditions ($p_{FWE} > 0.05$). We also found no significant change from placebo condition to citalopram condition in the difference in *Faces>Shapes* functional activations between block 1 and block 4 across the whole-brain ($p_{FWE} > 0.05$).

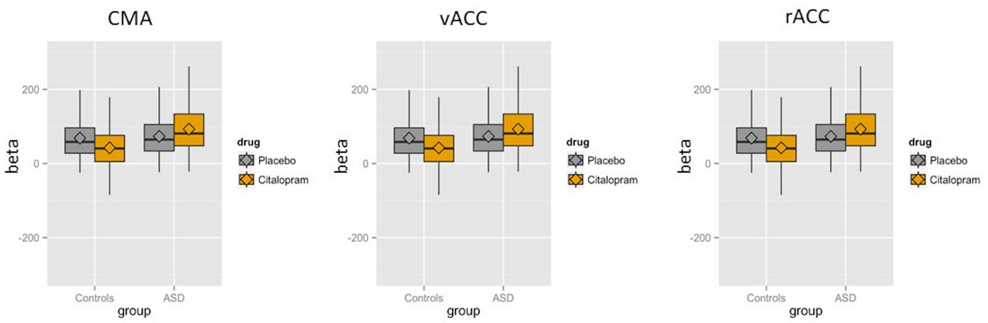

Supplementary Figure 1. **Average activations to negative facial emotions**. General activation differences in the contrast *Faces>Shapes* in the subregions of amygdala, ventromedial prefrontal cortex, and nucleus accumbens between individuals with and without autism spectrum disorder (ASD) in the placebo and citalopram conditions were investigated. There were significant group×drug interaction effects in the centromedial amygdala (CMA), rostral anterior cingulate cortex (rACC) and ventral ACC (vACC) ($b \geq 50.391$, $\chi^2 \geq 11.177$, $p_{corrected} \leq 0.030$). *Post-hoc* analyses revealed that group differences were not evident in placebo condition ($p>0.05$), but significant stronger activations in individuals with ASD than controls were observed in rACC and vACC ($b \geq 60.221$, $\chi^2 \geq 3.847$, $p \leq 0.050$). Among CMA, rACC and vACC, citalopram decreased activations in controls ($b \leq -29.031$, $\chi^2 \geq 5.943$, $p \leq 0.015$) but increased activations in ASD ($b \geq 21.360$, $\chi^2 \geq 5.160$, $p \leq 0.023$). None of the *post-hoc* analyses survived after correction for multiple comparisons ($p_{corrected}>0.05$).

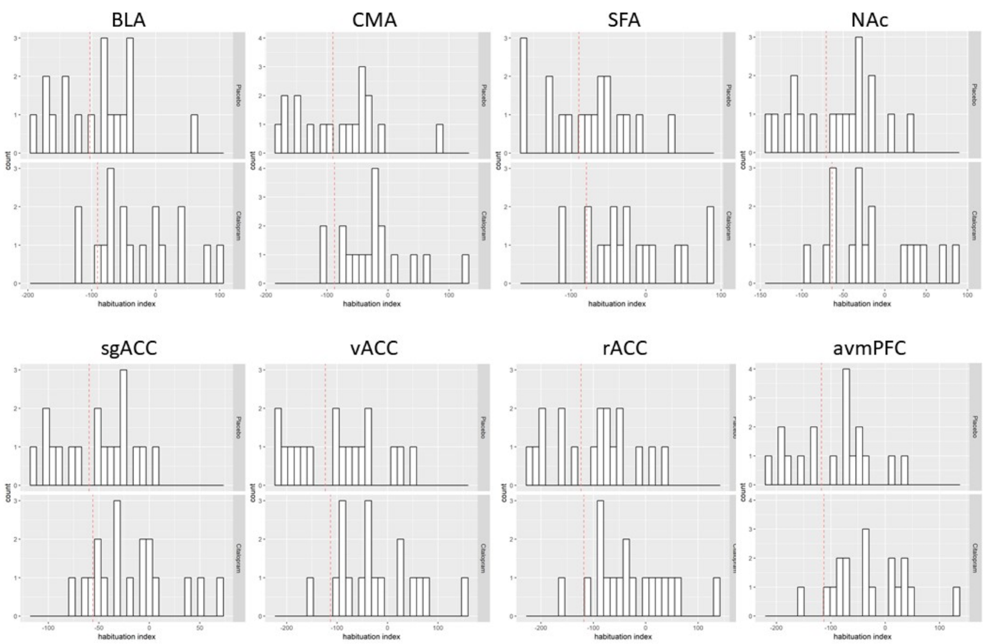

Supplementary Figure 2. **Histogram of habituation of autistic adults relative to control mean at baseline placebo condition and post citalopram.** The numbers of individuals with autism spectrum disorder (ASD) that had higher or lower habituation compared to the mean habituation in individuals without ASD in that condition (red dashed line) are visualised in histograms at baseline placebo condition (upper panel of each region) and post citalopram (lower panel of each region).
