# Supplementary material for: Serotonin differentially modulates the temporal dynamics of the limbic response to facial emotions in male adults with and without autism spectrum disorder (ASD): a randomised placebo-controlled single-dose crossover trial
Source: Neuropsychopharmacology. 2020 May 10;45(13):2248–56. doi: 10.1038/s41386-020-0693-0 (PMC7784897; doi:10.1038/s41386-020-0693-0)
Supplement: Supplementary file 2 — CONSORT Flowchart [file 41386_2020_693_MOESM2_ESM.doc]

**
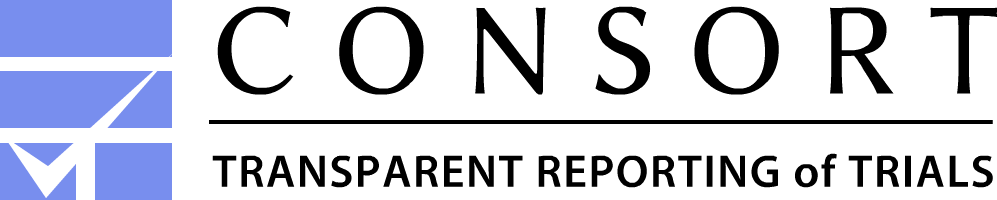
**

**CONSORT 2010 Flow Diagram**

**Allocation**

**Analysis**

**Follow-Up**

**Enrollment**

Assessed for eligibility (n=50)

Excluded (n=9)

♦  Not meeting inclusion criteria (n=9)

♦  Declined to participate (n=0)

♦  Other reasons (n=0)

Analysed (n=23)
♦ Excluded from analysis (give reasons) (n=0)

Lost to follow-up (give reasons) (n=0)

Discontinued intervention (give reasons) (n=0)

Allocated to placebo before citalopram (n=23)

♦ Received allocated intervention (n=23)

♦ Did not receive allocated intervention (give reasons) (n=0)

Lost to follow-up (give reasons) (n=0)

Discontinued intervention (give reasons) (n=1) *participant discontinued after allergic reaction to food colouring in the capsule*

pa

Allocated to citalopram before placebo (n=18)

♦ Received allocated intervention (n=18)

♦ Did not receive allocated intervention (give reasons) (n=0)

Analysed (n=17)
♦ Excluded from analysis (give reasons) (n=0)

Randomized (n=41)
